# Minimally Actuated Walking: Identifying Core Challenges to Economical Legged Locomotion Reveals Novel Solutions

**DOI:** 10.3389/frobt.2018.00058

**Published:** 2018-05-22

**Authors:** Ryan T Schroeder, John EA Bertram

**Affiliations:** ^1^ Biomedical Engineering Graduate Program, University of Calgary, Calgary, AB, Canada; ^2^ Cumming School of Medicine, University of Calgary, Calgary, AB, Canada

**Keywords:** bipedal locomotion, energetics, control optimization, dynamics modelling, work minimization

## Abstract

Terrestrial organisms adept at locomotion employ strut-like legs for economical and robust movement across the substrate. Although it is relatively easy to observe and analyze details of the solutions these organic systems have arrived at, it is not as easy to identify the problems these movement strategies have solved. As such, it is useful to investigate fundamental challenges that effective legged locomotion overcomes in order to understand why the mechanisms employed by biological systems provide viable solutions to these challenges. Such insight can inform the design and development of legged robots that may eventually match or exceed animal performance. In the context of human walking, we apply control optimization as a design strategy for simple bipedal walking machines with minimal actuation. This approach is used to discuss key facilitators of energetically efficient locomotion in simple bipedal walkers. Furthermore, we extrapolate the approach to a novel application—a theoretical exoskeleton attached to the trunk of a human walker—to demonstrate how coordinated efforts between bipedal actuation and a machine oscillator can potentially alleviate a meaningful portion of energetic exertion associated with leg function during human walking.

## 1. Introduction

The movement patterns of humans and other animals have been described in remarkable detail (Bregler et al., 2004; Winter, 2009). However, why any given movement pattern is used, and not some other, is currently not thoroughly understood. Some of the machinery of biological systems (aspects of their morphology and internal organization) is inherited (involving inevitable evolutionary inertia). As a result, it becomes a challenge to distinguish true adaptive design modifications that improve locomotory capability from adaptations that simply accommodate functionally neutral, or even detrimental, anachronistic features. This makes it very difficult to interpret the actions used in locomotion, regardless of the technical detail in which it is analyzed. It would be beneficial to put the actions observed in locomotion in the context of what they accomplish, and determine the advantages and limitations a particular strategy provides to the motor control system.

In this paper we describe our understanding of some key aspects regarding the dynamics of legged locomotion. This understanding has emerged largely from synthesizing the works of groups attempting to construct artificial walking machines. One advantage of trying to generate an original walking machine, rather than mimicking how humans or animals already move, is that it naturally identifies specific challenges and obstructions faced in legged locomotion without the biased expectations of an existing system. Identification of the challenges to be solved is one of the first steps in the design process and the discovery of new and different potential solutions.

There are two parts to this contribution. In the first part our intention is to describe an emerging perspective on legged locomotion dynamics. We use the context of human walking as a familiar example in which these ideas can be evaluated. The objective is to demonstrate how this perspective can aid in interpreting, and not just describing, observed movement strategies. In the second part of the contribution we explore the potential of simply actuated walking models to see how the identified challenges can be met most efficiently. Finally, we discuss the application of concepts on minimal actuation as an external environment (i.e., exoskeleton) for human locomotion by applying a theoretical oscillating impulse acting at the torso of a walking human.

In this contribution we discuss two hypotheses: (I) the action of the legs in human walking optimizes (or nearly optimizes) the interaction of the body mass with the external environment, and consequently specific movement strategies are selected based on taking advantage of energy saving opportunities while mitigating costlier alternatives; (II) external actuation applied directly to the center of mass (as opposed to at specific joints or in tandem with muscle groups along the body) can reduce the optimized leg work required in a reductionist bipedal optimal control model during walking. We advocate for a reductionist approach in our modelling in order to more clearly isolate features that contribute to effective actuation and control strategies. The proposal is that details of within leg function and other such physiology based features are secondary considerations relative to the more fundamental interaction between the body mass and its external environment that defines the task of locomotion.

## 2. Part I: Alternate Perspectives on the Task of Locomotion

One conventional definition of the task of locomotion might describe specific features observed in real-world examples (e.g., human walking can be distinguished from running because the latter has a non-contact phase during the stride cycle). However, in this case the solution to the problem is an observed feature without a clear definition of the problem being solved, so this approach mixes the task with the solutions implemented to accomplish the task. As such, it is nearly impossible to separate these two aspects of function, and this confuses the context of the observations and muddles our attempts to find and evaluate explanatory constructs.

Another common approach is to consider that locomotion simply seeks to transport the body from one location to another. However, this definition—fundamental though it may be—does not provide any real insight into how such a task should be managed. Indeed, one could imagine an infinite number of solutions to this formulation of the problem. In order to deal with this issue, we have recently proposed a reformulation of the fundamental task of legged locomotion (Croft et al., 2017). Briefly, any form of locomotion ultimately requires an interaction between the organism (more specifically, its mass) with its external environment. For example, steady level flight requires navigation of the body through the low density fluid of our atmosphere, while simultaneously balancing forces of lift and gravity, as well as thrust and drag. Given this fundamental task, there are a number of mechanisms potentially available to manage the body mass-environment interaction – fixed, rotary or flapping wings that can be powered by combustion engines, electric motors or muscles. In a similar manner, we contend that the fundamental task of legged locomotion should be considered the optimal dynamic interaction of the system mass with the external environment (e.g., in terrestrial locomotion, this is typically the substrate, gravitational force, etc.). An optimal (or near optimal) interaction allows for effective travel and must meet overarching goals determined by the priorities of the system (e.g., travelling some distance in a given amount of time, etc.) (Srinivasan and Ruina, 2006).

**Figure 1:**
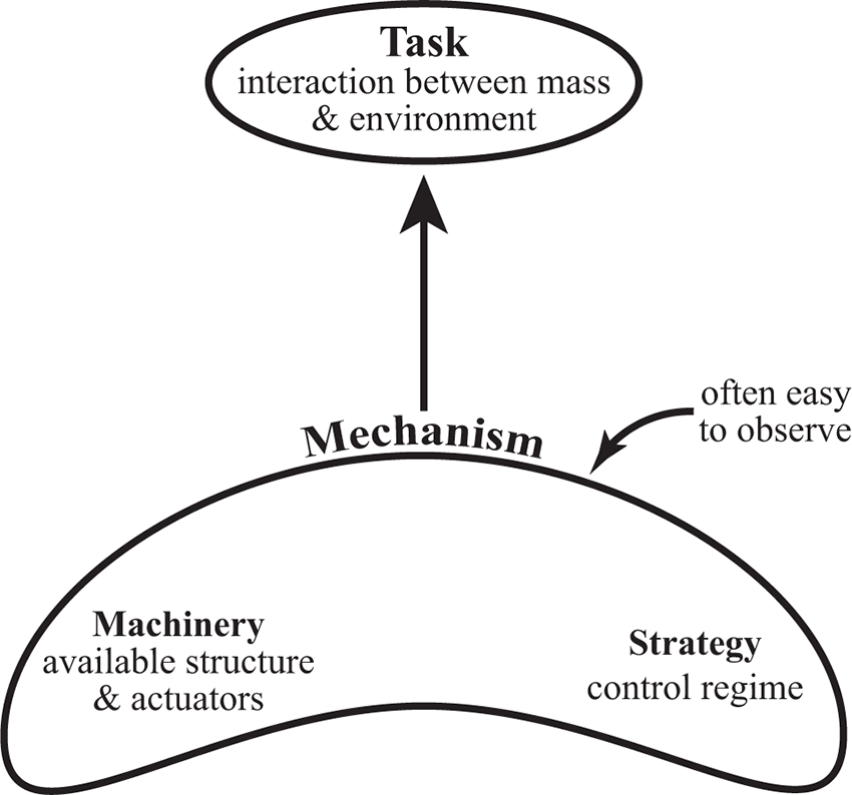
A diagram of the contextual hierarchy of locomotion. The task involves the fundamental optimal dynamic interaction of the body mass of the individual with the external environment through which they move. The task fulfills the goal of transportation as specified (distance, direction, speed, etc.), and one of the defining features in biological systems appears to be a drive towards energetic minimization. Mechanism(s) of locomotion (e.g., leg actuation, generation of joint torques, adjustment of leg stiffness, etc.) manage the task. The mechanisms available are constructed from the machinery (physical structures/tissues such as motors/muscles/skeleton) and the control strategy implemented to the machinery. Mechanisms tend to attract the attention of observers since the kinematics of the limbs are often readily visible, but the implementation of those mechanisms are only understood in the context of the fundamental task they accomplish.

Similar to flight, terrestrial locomotion has its own set of mechanisms that constitute the locomotory apparatus, all of which can be used to mediate the mass-environment interaction. The available mechanisms are composed of the machinery of the system (supporting tissues and actuators, whether organic or artificial) and the control regime implemented on the machinery (Figure 1). Still, the phrase optimal dynamic interaction remains ill-defined. In the following, we describe the role of energy minimization and analyze some basics of the human walking system while drawing on this perspective.

### 2.1. The Energetic Basis for Gait Parameter Selection

In natural human walking there is a standard, repeatable relationship between overground speed and stride frequency (Grieve and Gear, 1966; Bonnard and Pailhous, 1993). In fact, this relationship is so standard that it is possible to determine the bounds of normal walking and use these to define abnormal locomotion (Schwartz et al., 2008; Lythgo et al., 2011; Dixon et al., 2014). A different, but equally consistent relationship exists for human running (Kurz et al., 2005; Perry and Burnfield, 2010; Hein et al., 2012; Floría et al., 2018). However, documenting the gait parameters used in a given circumstance does little to explain why these are the particular movement strategies (nearly) universally selected. Certainly it is physically possible to walk (or run) with an extremely broad range of speeds, stride lengths or stride frequencies – so why is one set of solutions selected over others?

A hint at the basis for gait parameter selection (in this example, the parameters of interest are speed, step frequency and step length) and the natural constraints that determine the advantages of one strategy over another, can be drawn from the observation that individuals tend to choose a preferred walking speed when unburdened from explicit time constraints (e.g., rushing to catch the light at a crosswalk). Preferred walking speed tends to coincide with the global minimum cost of transport (CoT), or energy per distance traveled (Holt et al., 1995), although this observation continues to be challenged, (Godsiff et al., 2018). The CoT also appears to have an important influence on the selection of gait parameters over a range of walking speeds (Bertram and Ruina, 2001; Bertram, 2005). Since speed (*v*) is the product of step length (*d*_s_) and step frequency (*f*_s_), it is theoretically possible to manage any speed with an infinite number of step frequency-step length combinations. However, healthy humans tend to employ a generally standard relationship (Kuo, 2001).

As with the selection of preferred speed (and its step frequency-step length combination), the systematic change in these parameters from preferred speed can also be explained based on CoT energetics. As speed changes, step parameters (*d*_s_ and *f*_s_) are chosen to match the minimum solution for speed constraints on the objective function of CoT (Kuo, 2001). Although it may be suggested that speed change is a natural requirement of walking control, this result suggests that the control strategy is treated as a constrained optimization, where the optimization approaches the minimum cost combination available on the CoT surface.

**Figure 2:**
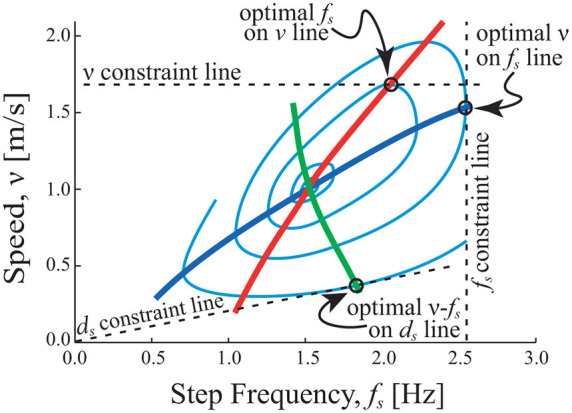
Constrained optimization of gait parameters in walking. Light blue contours represent equivalent cost combinations (iso-cost contours), where each contour is energetically less costly than the one residing outside it (minimum cost is central at the point where the red, blue and green lines intersect). For any constraint of speed (v), step frequency ($f_{s}$), or step length ($d_{s}$) the minimum cost solution features gait parameters where the constraint line is tangent to the cost contour (any other solution lies outside the contour, so is costlier). Constrained v relationship (red) is determined from horizontal tangents and the constrained $f_{s}$ relationship (blue) is determined from vertical tangents. The constrained $d_{s}$ relationship (green) is determined from sloped tangents (since $v=d_{s}f_{s}$).

Similarly, because speed is the product of step length and step frequency, it is also possible to demonstrate the constrained optimization response for the other two parameters (*d*_s_, *f*_s_) as well as for speed (*v*). When either step length or step frequency is constrained, the response of the other two parameters also tends to follow a minimum cost solution, but the solution differs based on the shape of the cost surface (Bertram, 2005). Optimizing the CoT surface as the objective function can explain a striking contrast in the speed-frequency relationship human subjects exhibit while walking with a constrained frequency (following a range of metronome beat frequencies), a constrained step length (walking in registry to a range of spaced floor markers) or a constrained speed (walking on a treadmill for a range of belt speeds) (Figure 2). This result shows that the selection of gait parameters in humans is not stereotyped but is actually quite plastic, and specifics of the gait are chosen (or at least highly influenced by a pressure) to minimize the cost of moving over the substrate. It should be acknowledged that other influences (e.g., obstacles to be avoided at the substrate, slippery surfaces, etc.) certainly play a role in the selection of gait parameters as well, and in fact, there is often an interdependence between other considerations and energy consumption (e.g., avoiding a slippery surface or else recovering from a fall has an energetic cost associated with it; Brandão et al., 2015). Regardless, in addition to human walking, energetic cost has also been shown to have a dominant influence on step width in human walking (Donelan et al., 2001), human running (Gutmann et al., 2006), walking in cats (Bertram et al., 2014) and for direct, acute manipulations of the objective function (the CoT surface; Selinger et al., 2015).

### 2.2. Actuator Performance

The evidence above indicates that minimizing energy expenditure is a key control factor in humans (and likely in other animals as well). So it might be useful to consider *how* energy can be minimized. One option is to seek more efficient actuators. However, even if ideal efficiencies are possible, this approach has a yield limited by the cost of the strategy. However, the strategy itself can be modulated (adjusting the control regime, Figure 1) and such modulation can have a substantial consequence for cost. Consider, for instance, that most high fidelity legged robots, such as Honda Asimo, have motors that are at least 3–5 times more efficient than mammalian muscle, yet their CoT for walking on legs can be well over 10 times greater than that of humans (Collins et al., 2005). Understanding the subtleties of human walking control may have large payoffs in robotics.

In many engineering circumstances inadequate energy or power capabilities can be addressed with the implementation of more sophisticated actuators and/or larger power supplies. However, state-of-the-art technology capable of maximizing performance potential is often very expensive. Furthermore, scaling up the power of actuator systems typically comes at a trade-off of increased volume and weight not particularly suitable for the mobility desired in locomotion systems. Thus, artificial design options may be informed by an understanding of how organic systems manage impressive performance despite efficiency limitations. In this, we contend that the goal of energy minimization directs attention to some important factors influencing general performance of legged locomotion systems and the effective movement strategies available to them.

### 2.3. Energy Transduction in Walking

The predominant conventional approach to analysis of walking gaits considers transduction of energy forms as it flows within the system (e.g., between potential and kinetic energy; Cavagna et al., 1977; Cavagna et al., 2002). However, we argue that a more comprehensive strategy should also track energy flow into and out of the system (Srinivasan and Ruina, 2007). This aspect is important because energy loss must be paid back in the form of mechanical work, and this imparts a metabolic cost on the organism, at least for the case of a steady state gait. Thus, assuming energy loss is undesirable, the manifestation of this loss must indicate either a limitation of the specific gait mechanism used and/or a constraint that restricts the strategy chosen. Understanding how the loss occurs (and why it occurs) allows for clear distinction of various strategies available to manage the interaction with the substrate. How does energy move through a legged walking system?

**Figure 3:**
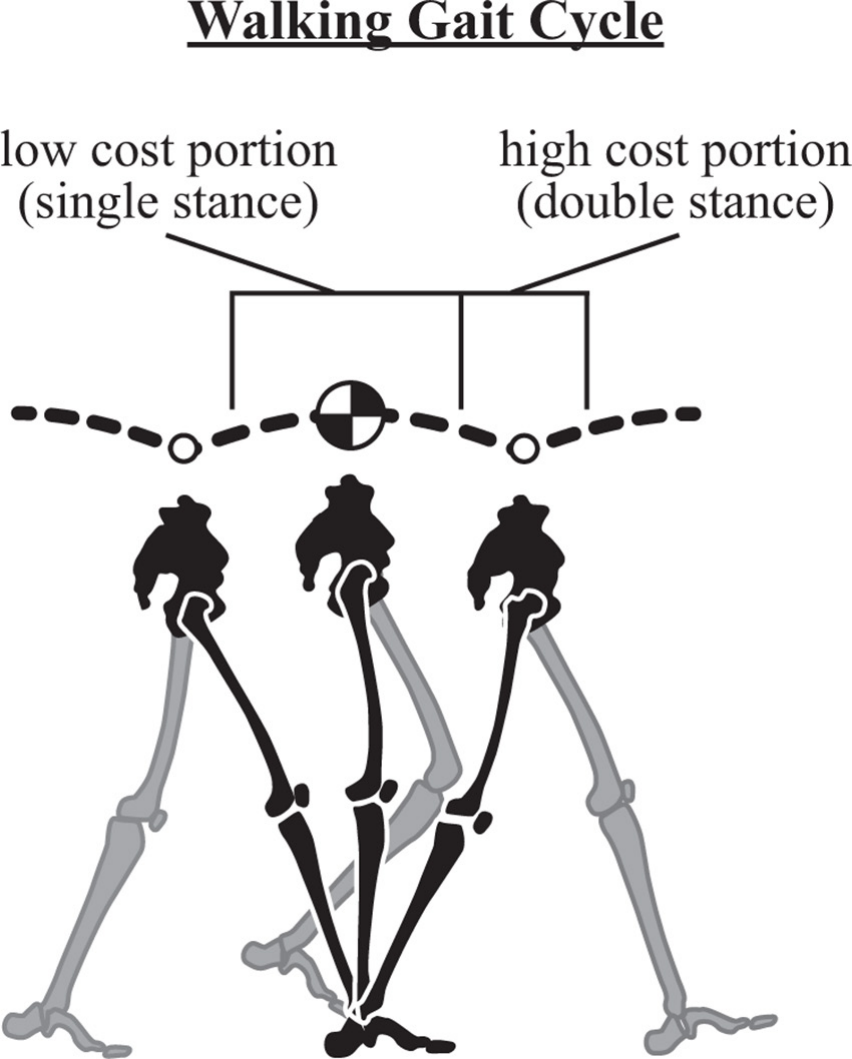
The walking gait cycle. The walking stride involves a low energetic cost portion where gravity redirects the center of mass from upward to downward (a passive transition) – this occurs during single stance in the inverted pendulum phase of walking. The stride also involves a high cost portion where the center of mass is redirected from downward to upward (active). This is costly because it must be mediated by action of the legs. Note vertical fluctuations in the trajectory are slightly exaggerated for clarity.

Walking is commonly described based on variations of an inverted pendulum model where potential (PE) and kinetic energy (KE) fluctuations are largely out of phase during the single stance portion of the stride. During this time, the center of mass (CoM) rises to a maximum (PE increases as KE decreases) and then begins to fall (PE decreases as KE increases), and this passive redirection is largely managed by the acceleration of gravity. Direct exchange of PE and KE during single stance implies a near constant total mechanical energy and minimal energetic losses from the system (i.e., single stance represents a low cost portion of the gait cycle; Figure 3). Typically, the inverted pendulum model only considers the stance phase described, and as such, energy losses from the system are often neglected, even though they do occur in real-world locomotion.

Specifically, redirection of the CoM (from down to up) incurs a cost that must be mediated by the action of the legs (Srinivasan and Ruina, 2007). This occurs during double stance in walking when the CoM reaches its lowest point in the gait cycle. Since this vertical redirection is largely active, it requires a high energetic cost (relative to the rest of the gait cycle; Figure 3), which manifests as a loss of energy that must be repaid through leg work (to maintain steady state gait). It is informative to look more closely at the mechanisms through which this can occur in walking, and consider strategies implemented to minimize the energetic cost.

### 2.4. Collision Dynamics and Transition Loss

An important, and often overlooked, cause of energy loss originates with collision dynamics. A collision involves an abrupt change in the momentum of a body when it interacts with an impulsive force, and this results in a loss of energy. In terrestrial locomotion the legs contact the substrate and alter the trajectory of the individual’s mass. Although in biological systems these interactions may not appear particularly impulsive in the classical dynamics sense, the trajectory change of the body mass from downward to upward during the step-to-step transition of walking can be viewed in terms of collision events (Kuo, 2002; Kuo et al., 2005; Ruina et al., 2005; Srinivasan and Ruina, 2006; Lee et al., 2013). The organism experiences a loss of kinetic energy as the ground reaction force does mechanical work on the CoM (in addition to the trunk, this also includes body segments with motion relative to the trunk). The energetic consequence on the organism can be quite meaningful and is quantified by the dot product of the ground reaction force (GRF) vector and the CoM velocity vector integrated over the duration of the impulse (Lee et al., 2011). The consequence of this relationship is such that a perpendicular vector orientation results in no work done by the impulse (no energy loss), since cosine of 90° (and 270°) equals zero. However, non-zero mechanical work is done with any other vector geometry.

Although in terrestrial locomotion the limbs act primarily as struts, the inherent compliance of the jointed limb means that force application is not purely impulsive but is instead distributed over the duration of the step. Nevertheless, the basic principles that govern redirection of colliding objects can be applied to the redirection of the CoM during locomotion. This results in an energy loss that forms the basis of legged locomotion costs in gaits such as walking and running. An alternative view is that at least some energy is retained and recovered by elastic structures in the leg. Elastic energy recovery is undoubtedly useful, but it is not essential to gait (Srinivasan and Ruina, 2006). The optimal CoM path appears to be identical whether the supporting legs have elasticity or not (Ruina et al., 2005). In reality, it is likely that collision mitigation and elastic energy recovery occur – with both being complimentary (Bertram and Hasaneini, 2013).

### 2.5. Minimizing Energy Loss at the Step-to-Step Transition

The reader may recall that the high cost portion of walking occurs when the CoM is redirected from moving downward to upward at the step-to-step transition during double stance (Figure 3) and forward momentum is maintained over the stride cycle. Since there are two legs contacting the substrate over the transition, various strategies exist to mitigate energy loss if the two limbs work together in a coordinated manner.

**Figure 4:**
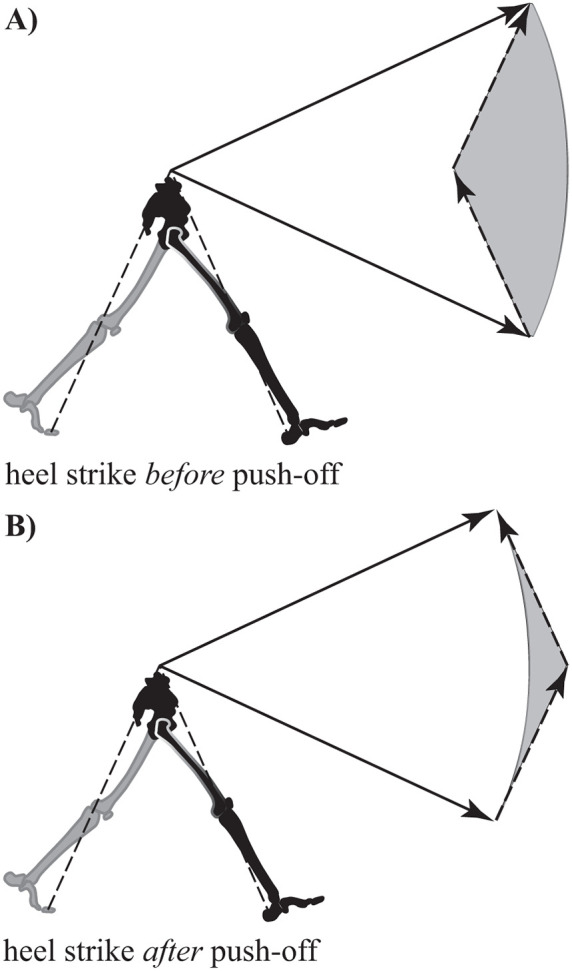
Reorientation of the center of mass velocity vector during the step-to-step transition (double stance) in walking. **(** **A** **)** Energy inefficient walking – the leading leg makes contact (heel strike) and the velocity vector magnitude decreases along a path parallel to the contact leg (collision loss). The trailing leg then applies a push-off force accelerating the velocity vector back to its original magnitude. The circular arc connecting the tips of the velocity vectors indicates a change in vector orientation without change in magnitude (constant kinetic energy). The area between the arc and the vector path during the step-to-step transition (shaded grey) is related to the work required for the transition. **(** **B** **)** Energy efficient walking – a preemptive push-off occurs from the trailing leg prior to heel strike of the leading leg. The push-off shifts the velocity vector to a more horizontal orientation making the interaction between the new stance leg and velocity vector much more favorable (less work required in the transition is indicated by the reduced size of the grey shaded area). Note leg and velocity vector angles are exaggerated for clarity.

In fact, details of the step-to-step transition turn out to be critically important in determining the overall CoT of bipedal walking (Donelan et al., 2002a). One option is to use heel contact at the beginning of stance to redirect the CoM, where it is simply vaulted over the strut-like leg (Figure 4A). However, this vaulting action inevitably results in energy loss as the strut redirects the path of the CoM. This loss can be replaced by push-off work from the trailing (former) stance leg, which momentarily maintains ground contact during the transition period.

Although a strategy utilizing heel strike *before* push-off is a viable solution, it is not the most effective strategy for managing the step-to-step transition. Instead, it is highly advantageous to initiate heel strike just *after* push-off from the trailing leg (Kuo, 2002; Donelan et al., 2002b; Kuo et al., 2005). This particular sequencing allows the previous stance limb to begin redirecting the CoM with a forward and upward impulse (commonly referred to as preemptive push-off) before the collision occurs. The preemptive push-off helps to orient the CoM velocity vector more perpendicular relative to the force vector resulting from heel strike (Figure 4B). Ultimately, this allows for substantial reduction of momentum (and energy) loss due to the collision (Ruina et al., 2005).

It is possible to eliminate collision loss at the step-to-step transition with a gait sometimes referred to as Groucho walking. To accomplish this, the substrate is contacted with a relatively straight leg that initially flexes and then extends over stance. This allows the CoM to maintain its vertical position as it passes over the contact point in a straight horizontal path. Although this can eliminate the collision-based loss, it turns out that the leg work required (extending and flexing under the load) is greater than the collision loss it prevents. This has been shown both analytically (Ruina et al., 2005; Gordon et al., 2009) and empirically (Ortega and Farley, 2005; Gordon et al., 2009).

Another feature of an energy effective step-to-step transition involves swing leg retraction. In swing leg retraction the impending next stance leg is accelerated opposite the direction of travel just prior to it contacting the ground (heel strike). Due to mechanical coupling of both legs at the pelvis, rearward acceleration of the leading leg results in a reaction force (at the hip) that accelerates the rest of the body forward, and this aids push-off of the trailing leg. As such, impulses from push-off can be partially down regulated. However, the relative magnitude and timing of stance leg preemptive push-off and swing leg retraction requires coordination to optimize energetic cost (Hasaneini et al., 2017).

In natural human gait, an optimal step-to-step transition strategy comprises a trade-off between collision loss reduction and leg work associated with flexion and extension at the joints. (Bertram and Hasaneini, 2013). It should be emphasized, however, that an effective step-to-step transition in walking requires coordination between both legs in the approach up to and during the transition. This coordination is indicated by the distinctive double hump vertical GRF of human walking. Whereas this pattern is generally interpreted with regard to function of each leg individually, it occurs largely because the second vertical maxima in stance is associated with the critical preemptive push-off while the first indicates the transfer of load to the new stance leg (i.e., heel strike). Each portion of the contact should be functionally interpreted with respect to its role in the transition, rather than as an aspect of the force sequence an individual leg generates over stance (Usherwood, 2016; Bertram, 2016c).

Given some insight into the subtle strategies available to manage the energetic cost of the step-to-step transition in human walking as described above, how can this be applied to alternative designs in legged robots? Passive dynamic walking machines (no actuators nor controllers, as the name implies) are equipped with legs that spontaneously swing in an appropriate manner to stabilize forward progress (McGeer, 1990) while moving down a slightly sloped ramp. With each step, a small amount of PE is converted to KE as the machine falls forward, however this extra energy is soon lost due to collision interactions with the ramp’s surface at the step-to-step transition (Garcia et al., 1998). Ultimately, this allows for a near steady state gait pattern that qualitatively looks remarkably like human walking (Bertram, 2016a).

Variations on the passive dynamic walker incorporate simple actuators that can provide small impulses at each leg to allow for level surface walking (Collins et al., 2005). As discussed above, the preemptive push-off impulse of the actuator plays an important role in overcoming energetic losses due to collisions while redirecting the CoM from a downward trajectory to upward.

There is also a secondary role of the active (preemptive) push-off in that it helps facilitate the leg’s forward swing in order to set up the next step. Ankle plantar flexion just prior to heel strike has been associated with preparing the leg for the swing portion of the step (Winter and Robertson, 1978; Meinders et al., 1998). It is likely that the push-off does indeed fulfill this functional role, but the swing preparation and preemptive, collision mitigating push-off are not mutually exclusive, so it is likely that both roles are satisfied by this single action (Zelik and Adamczyk, 2016).

## 3. Part II: Simply Actuated Walking Models

In this part of the contribution, we outline various options for reductionist bipedal designs that rely (to varying degrees) on many of the concepts discussed in Part I. We begin with single actuator mechanisms and progress to multi-actuator mechanisms, in order to alleviate some of the restrictive dynamics inherent in simpler designs. Finally, we discuss an application of similar concepts to an exoskeleton strapped to the trunk of a walking human. For most of the models presented, control optimization software is used to determine energetically minimal solutions. These solutions are then analyzed post-hoc in order to isolate important features that either support or violate expectations of what economical locomotion should look like based on an understanding of established theory. This section is organized with specifically chosen models to invoke a discussion about important dynamic restrictions and the consequences of different actuation patterns on the energetics of effective locomotion during bipedal walking. A primary objective of the models is to explore the limit of reducing the number of actuators necessary to allow active bipedal locomotion (at least in the planar case).

### 3.1. Single Actuator Designs

#### 3.1.1. Constant Force Single Actuator Inverted Pendulum

**Figure 5:**
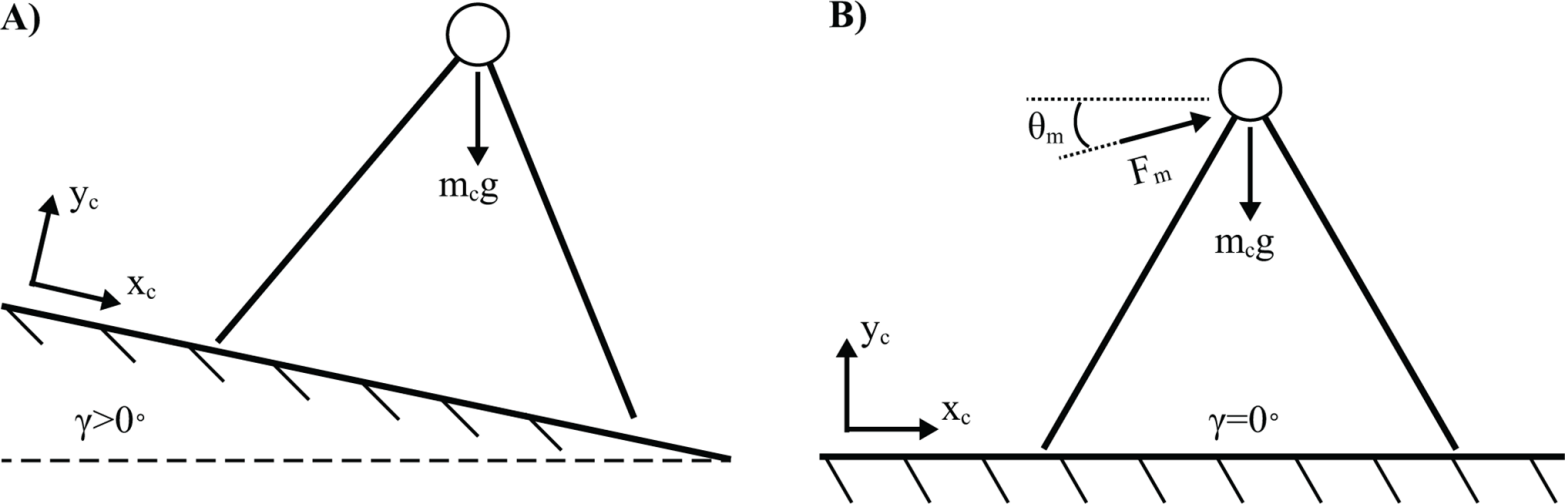
Constant force actuator drives a quasi-passive dynamic walker. **(** **A** **)** Passive dynamic walker on sloped ground. **(** **B** **)** Quasi-passive dynamic walker equivalent to (A) with a constant force actuator on flat ground.

The placement of an actuator at each leg to power foot extension is one means by which to add work and replace energy lost from collisions and other inefficiencies (Collins et al., 2005). This may be considered a bioinspired design, but it is likely that much of the energetic benefit is achievable merely with a single actuator acting directly at the CoM. In fact, it is possible to mathematically replicate the constant gravitational forces acting on the passive dynamic walker on a sloped surface with a single actuator (constant orientation and force magnitude) acting directly on the CoM for a walker on a level surface (Figure 5). To solve for the actuator orientation and magnitude, the gravitational force (acting on a reference frame of an elevated slope, $\gamma >0^{\circ }$) is set equal to a constant actuator force plus a gravitational force (acting on a reference frame of no slope, $\gamma =0^{\circ }$). Two equations are formulated for the forces in the horizontal (Eq. [1]) and vertical (Eq. [2]) directions (left side of the equations: gravitational force acting on a sloped surface, right side of the equations: gravitational and actuator forces acting on a flat surface).

(1) $$m_{c}g\cos\left(1.5\pi +\gamma \right)=0+F_{m}\cos\left(\theta_{m}\right)$$

(2) $$m_{c}g\sin\left(1.5\pi +\gamma \right)=-m_{c}g+F_{m}\sin\left(\theta_{m}\right)$$

where $m_{c}$ is the body mass, g is gravitational acceleration (e.g. $9.81\frac{m}{s^{2}}$), $F_{m}$ is a constant actuator force, $\theta_{m}$ is the angle of the actuator and γ is the angle of the ground’s slope. When equations [1,2] are solved simultaneously, $F_{m}$ and $\theta_{m}$ are analytically determined.

(3) $$F_{m}=m_{c}g\sqrt{2+2\sin\left(1.5\pi +\gamma \right)}$$

(4) $$\theta_{m}=\frac{\gamma }{2}$$

The strategy of powering a walking machine purely with gravitational forces means that no batteries are necessary, and the work done by gravity is essentially free. Furthermore, only a very subtle slope is needed to overcome the energy losses due to collisions if the system is constructed properly. However, the constant-force actuator alternative must do work to mitigate gravitational forces as well as overcome collision losses. Although this actuation strategy may exist as a viable solution, the constant force profile can likely be improved upon. For example, more sophisticated strategies might leverage dynamic force production as a means for reducing the mechanical work done by the actuator.

### 3.1.2. Optimized Single Actuator (Horizontal) Inverted Pendulum

**Figure 6:**
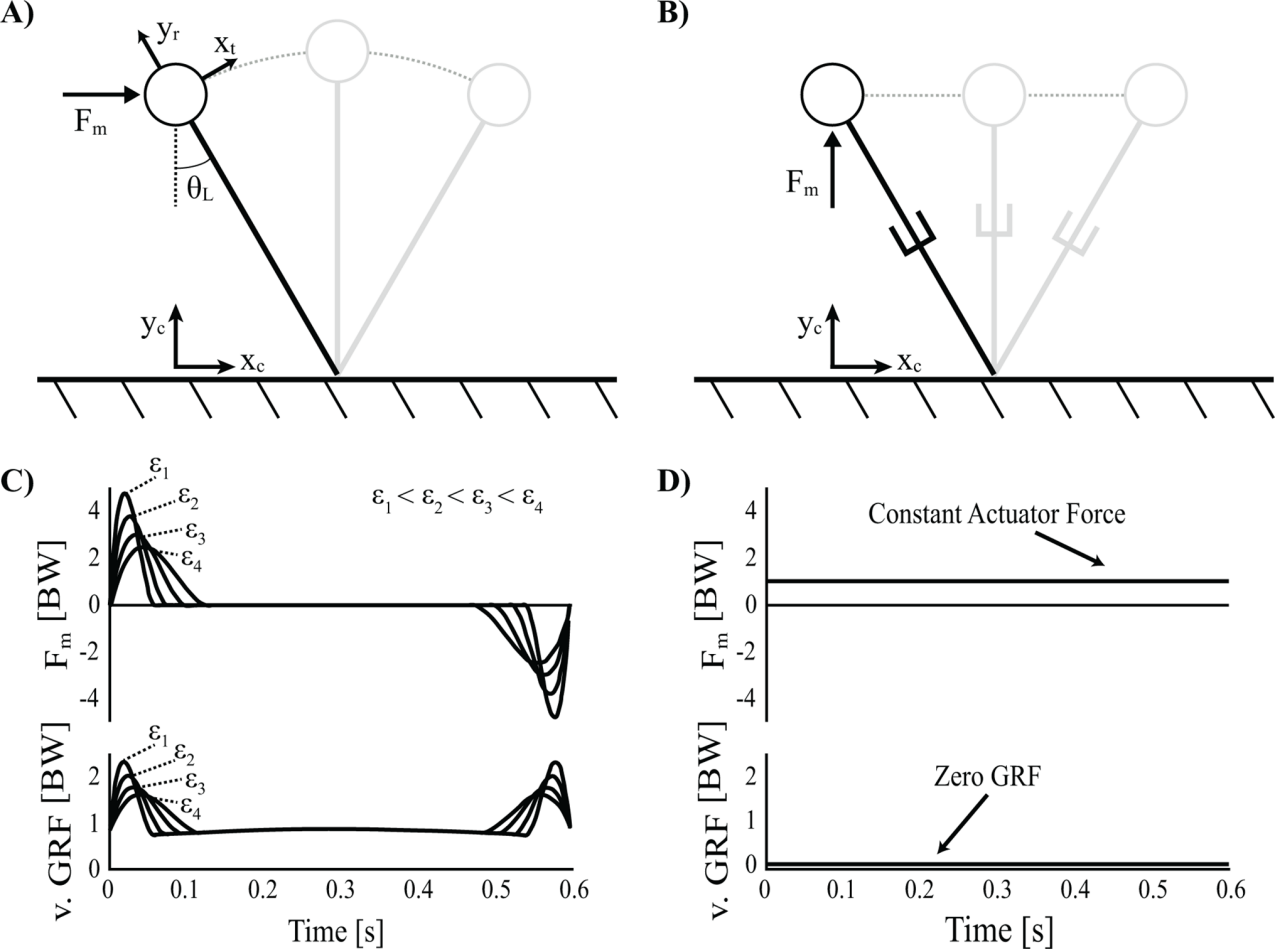
Single actuator walking models. **(** **A** **)** Inverted pendulum control optimization model with a horizontal actuator and rigid legs. **(** **B** **)** Groucho walker with a vertical actuator and collapsible legs. **(** **C** **)** Vertical ground reaction force (v. GRF) and actuator force ($F_{m}$) plotted over time, in units of body weights (BW) for the optimal solution of the inverted pendulum model with a horizontal actuator (multiple force rate scaling constants are shown:  ($\epsilon_{1}=3x10^{-6},\epsilon_{2}=9x10^{-6},\epsilon_{3}=3x10^{-5}\;and\;\epsilon_{4}=9x10^{-5}$). (**D)** The Groucho walker with a vertical actuator has zero vertical GRF and a constant $F_{m}$ that defaults to supporting body weight.

Assuming that ideal actuation strategies are unknown a priori, control optimization procedures can be used to determine the actuator force profile that minimizes mechanical work over a step. Although a specific actuator angle ($\theta_{m}=\frac{\gamma }{2}$) was necessary to replicate the gravitational forces acting on a passive-dynamic walker down a slope, this angle is not required for a non-constant actuator force profile. Instead, a fixed horizontal orientation ($\theta_{m}=0^{\circ }$) was chosen somewhat arbitrarily (Figure 6A), although this configuration does allow for symmetrical force profiles mirrored about mid-stance (i.e., when the CoM is directly above the foot-ground contact). The equation of motion for a standard inverted pendulum model is expanded to reflect the influence of a fixed horizontal actuator.

$$m_{c}{\ddot{x}}_{t}=m_{c}g\cos\left(1.5\pi -\theta_{L}\right)+F_{m}\cos\left(-\theta_{L}\right)$$

(5) $$-m_{c}{\ddot{\theta }}_{L}L=m_{c}g\cos\left(1.5\pi -\theta_{L}\right)+F_{m}\cos\left(-\theta_{L}\right)$$

where ${\ddot{x}}_{t}$ is the tangential acceleration of the CoM motion and $\theta_{L}$ is the leg angle relative to vertical. Note, the actuator force, $F_{m}$, is not constant as in Eq. [3], however it is a control variable optimized in the control optimization process. The reaction force of the rigid leg is also shown for the inverted pendulum.

$$R_{r}=m_{c}{\ddot{y}}_{r}-m_{c}g\sin\left(1.5\pi -\theta_{L}\right)-F_{m}\sin\left(-\theta_{L}\right)$$

(6) $$R_{r}=-m_{c}{\dot{\theta }}_{L}^{2}L-m_{c}g\sin\left(1.5\pi -\theta_{L}\right)-F_{m}\sin\left(-\theta_{L}\right)$$

where L is a constant leg length used in the model. Gait parameters such as average forward velocity (v), step frequency ($f_{s}$) and step length ($d_{s}$) are all pre-determined constraints in the model. Specifically, time is constrained from initial point $t_{o}=0$ to final point $t_{f}=T_{s}$ where $T_{s}=\frac{d_{s}}{v}$. Step length was enforced by constraining CoM position at the initial point $x_{c}=0$ and at the final point $x_{c}=d_{s}$. A biologically realistic step length was chosen (Alexander, 1992) for an average forward velocity of $v=1\frac{m}{s}$.

(7) $$d_{s}=1.25\left(\frac{L_{max}^{0.7}}{g^{0.3}}\right)v^{0.6}$$

A path constraint was applied to the optimization in order to ensure that only solutions requiring reaction forces greater than or equal to zero (i.e. $R_{r}\geq 0$) throughout the step were considered (tension leg forces were not allowed since this would require the foot to actively stick to the ground). Endpoint constraints were also applied such that only periodic force profiles and CoM kinematics (i.e. steady state patterns) were considered.

Finally, the objective function, or cost function, was chosen to minimize the summation of a mechanical work-based cost and a force-rate-squared term (scaled by an arbitrarily small number, $\epsilon_{1}$). The force-rate-squared term was employed in order to avoid extreme impulsive actuator forces (a theoretical, but unrealistic optimum). This allows for smoother force profiles and a quicker optimization with more reliable results. The cost function is explicitly stated.

(8) $$C=\int_{t_{o}}^{t_{f}}\left({\dot{W}}_{m}^{+}-{\dot{W}}_{m}^{-}+\epsilon_{1}{\dot{F}}_{m}^{2}\right)dt$$

where ${\dot{W}}_{m}^{+}$ is the positive mechanical power of the actuator, ${\dot{W}}_{m}^{-}$ is the negative mechanical power of the actuator and ${\dot{F}}_{m}$ is a time-rate of the actuator force. Mechanical power is calculated.

$${\dot{W}}_{m}=\;F_{m}{\dot{x}}_{t}\cos\left(-\theta_{L}\right)={\dot{W}}_{m}^{+}-{\dot{W}}_{m}^{-}$$

(9) $${\dot{W}}_{m}=F_{m}{\dot{\theta }}_{L}L\cos\left(-\theta_{L}\right)={\dot{W}}_{m}^{+}-{\dot{W}}_{m}^{-}$$

Orthogonality of ${\dot{W}}_{m}^{+}$ and ${\dot{W}}_{m}^{-}$ was ensured by augmenting the cost function with an additional cost term scaled by a small number: $\epsilon_{o}{\dot{W}}_{m}^{+}{\dot{W}}_{m}^{-}$. The cost of this term was always driven to zero in all optimizations, and therefore it did not contribute to the overall cost of the solution. However, its implementation ensures that the actuator can never produce both positive and negative work simultaneously.

A sparse nonlinear optimizer program (SNOPT) (Gill et al., 2005) was used to solve for the optimization problem and the MATLAB (The MathWorks Inc. in Natick, Massachusetts) software GPOPS-II (Patterson and Rao, 2014) was used for problem discretization and setup. A duel part optimization process was employed. In the first part, multiple solutions (*n* = 15) were determined with random initial guesses in order to reduce the likelihood of settling at a local optimum in the cost function. The lowest cost solution of the 15 random initial guesses (i.e., seed) was then put through a perturbation phase where initial guesses were supplied by the seed solution plus random noise scaled to 12.5%, or one eighth, of each variable’s overall range. Multiple perturbation solutions (*n* = 15) were determined, and the seed solution was only considered optimal if its cost remained lower than the outcome of all perturbation iterations. In the case that a perturbation iteration resulted in a lower cost solution, it was chosen as the new seed, and an additional round of perturbation iterations was conducted. This process was reiterated until the seed’s cost was found to be lower than all perturbation solutions. The perturbation phase was conducted in order to fine tune the optimal solution.

The solution resulting from the optimization is characterized by an actuation strategy similar to what optimal control theorists often refer to as bang-coast-bang (Athans and Falb, 1969). Specifically, near impulsive forces mark the beginning and end of the step, with a quiet period of inactivation toward mid-stance ($t_{o}=0$ is associated with the beginning of stance, essentially heel strike). The first bang (impulse), toward the beginning of the step, is positive (i.e., in the direction of travel) and accelerates the body’s tangential motion from rest. The second bang, toward the end of the step, is negative (i.e., opposite the direction of travel) and decelerates the body’s tangential motion back to rest (Figure 6C). It should be noted that a true bang-coast-bang pattern more commonly exhibits instantaneous discontinuities of state, however this kind of solution is penalized with the force-rate-squared term. Nevertheless, the near impulsive forces (high magnitude, short duration) can still be considered an approximation of a more literal bang-coast-bang pattern. To illustrate the smoothing effect of the force rate cost, the optimization was run with force rate scaling constants over a broad range of values ($\epsilon_{1}=3x10^{-6},\epsilon_{2}=9x10^{-6},\epsilon_{3}=3x10^{-5}\;and\;\epsilon_{4}=9x10^{-5}$) As the scaling constant increases, the force magnitudes decrease and are spread out over a longer period of time in order to achieve the impulse required by the solution (Figure 6C).

One can compare the dynamic function of the optimization’s near impulsive forces to similar actions in human walking: push-off and heel strike, respectively. In efficient bipedal locomotion, the preemptive push-off earns its name by initiating the impulse just before heel strike. As a result of adding energy into the system first, the CoM velocity vector is redirected upwards (and forwards). This serves to orient the angle relating force and velocity vectors more perpendicularly, and ultimately results in a reduction of collision losses imparted by the heel strike impulse (Figure 4B).

However, the current walker utilizes a reversed strategy with a heel strike-like impulse toward the end of the step to slow to a stop and then a push-off-like impulse toward the beginning of the next step to accelerate back to speed again. This strategy is particularly expensive and re-emphasizes the benefit of optimal sequencing of leg forces during human walking. The reason the walker cannot utilize the alternate beneficial sequencing is because it must satisfy constraints of periodicity. The result is that the CoM is required to begin and end with zero velocity at the stepping transition, as a direct result of the inverted pendulum beginning with a rising arc and ending with a falling arc. As such, a unique continuous periodic solution exists where the CoM begins and ends with zero velocity (note the option of a collisional impulse at the transition is excluded since it creates a discontinuity in the CoM trajectory).

### 3.1.3. Single Actuator Groucho Walker

An alternative system which allows for radial deviations in the CoM (e.g., telescopic legs) could potentially achieve continuous periodic gaits. Such a system might rely on a vertically oriented actuator in order to effectively support the weight of the body, since the legs are not actuated and cannot bear load (Figure 6B,D). In this case, it is easy to imagine that a trivial solution would be optimal. Specifically, the solution could utilize a constant force actuator to consistently support body weight along a straight path. Further, because no vertical oscillation is necessary, zero mechanical work is required of the actuator.

It should be noted that the analogous gait in human walking—referred to earlier in Part I as Groucho walking (Bertram et al., 2002)—imparts a much greater cost on the person relative to natural walking (Ortega and Farley, 2005; Gordon et al., 2009). This has a very different energetic consequence compared to that of the isometric force actuator, simply because the actuator is supporting body weight from an ideal orientation underneath the body. Essentially, this solution represents the dynamic equivalent of a wheel, which allows for continuous support even as it rolls in a straight path along the ground. Another example of such a system is the gliding of an ice skater. The legs simply bear the weight of the body but do no work to displace the body.

Perhaps a system utilizing a vertically oriented actuator might take advantage of the rigid strut-like leg in the inverted pendulum and use the actuator to provide impulses at the stepping transition. Although such a walking mechanism is theoretically possible, there is little the actuator could do without requiring a tension force in the leg to keep it grounded, or else launch itself into the air during actuation.

In the following section, we discuss the potential of walking robots that require multiple actuators to accomplish efficient walking gaits.

### 3.2. Multiple-Actuator Designs

#### 3.2.1. Inverted Pendulum with Telescopic Leg Actuators

**Figure 7:**
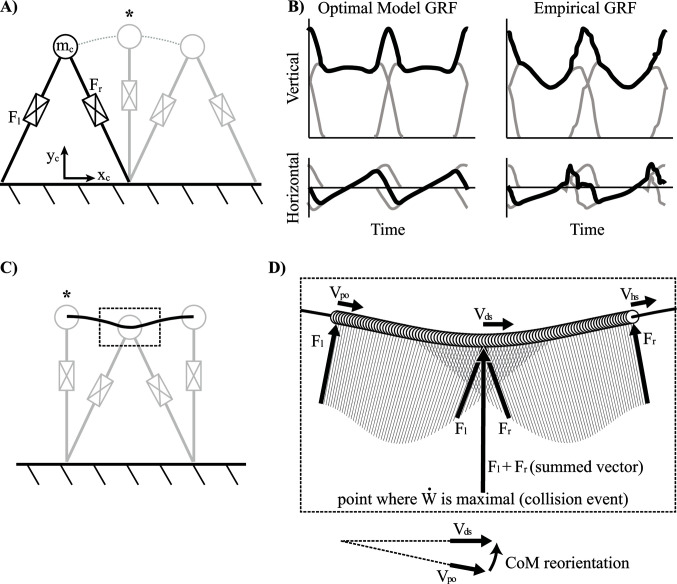
Inverted pendulum model with telescopic leg actuators. **(** **A** **)** Kinematics of center of mass (CoM) trajectory and legs shown for double stance, mid-stance (asterisk) and double stance again. **(** **B** **)** Two consecutive cycles of vertical and horizontal ground reaction forces (GRF) are shown as outputs of the optimization model alongside empirical force plate data of human walking [unfiltered force record, after Bertram (2016b)]. Force data from individual legs are shown in grey whilst the total force is shown in black. **(** **C** **)** Kinematics of CoM trajectory are shown for mid-stance [asterisk notes mid-stance as a common point in the gait cycle between panes (A) and (C)], double stance and mid-stance again. The dashed box indicates the step-to-step transition region shown in **(** **D)** where the CoM velocity vector is reoriented from the beginning of push-off ($V_{po}$) through to the middle of double stance ($V_{ds}$) and to the end of heel strike ($V_{hs}$). Force vectors of both legs  ($F_{l}$ and $F_{r}$) and the CoM are shown for multiple snapshots over the transition (thin lines are sequential vectors over the transition). The point of maximal mechanical power is shown at the middle of double stance where the legs do positive and negative work simultaneously, even though the summed vector appears perpendicular to the CoM velocity vector (misleadingly implies zero work).

The fully passive inverted pendulum model has been used to characterize the fundamentals of human walking for many decades (Cavagna and Margaria, 1966; Alexander, 1980). Although it remains a successful model for describing aspects of natural gait, it is limited by its capacity to predict motor responses during atypical walking gaits. Here, the word atypical specifies any such gait where the inverted pendulum is not naturally selected (e.g., Groucho walking, running, skipping, etc.). This is somewhat peculiar given that all forms of typical *and* atypical gaits still utilize the same morphological leg. Thus, an alternative way to think about the inverted pendulum is as a motor control strategy for effective bipedal walking. Specifically, it is the minimal energetic cost associated with the distinctive arced trajectory of the inverted pendulum that allows for efficient bipedal walking. Although focus is generally on the minimal work required for the inverted pendulum during single stance, a bipedal system *does* require an instantaneous impulse to redirect the CoM from downward to upward at the step-to-step transition (assuming a steady, periodic gait), and this impulse *does* impart a quantifiable cost on the system. Of course, in reality, the biological biped does not utilize ideal impulses (instantaneous with infinite magnitude), but rather, it imparts impulse-like forces (high magnitude, relatively short burst duration) to manage CoM redirection. These impulsive forces largely align with the orientation of the legs in the form of a push-off and a heel strike force, which both contribute to the characteristic double-humped profile of the vertical ground reaction force, as discussed in Part I (Figure 7B).

To test whether these dynamics are optimal without explicitly constraining them (such as with the inverted pendulum model), two telescopic legs with linear actuators are utilized to provide optimized force profiles that manage the CoM trajectory with minimal mechanical work. A similar model was utilized by Srinivasan and Ruina (2006). Even though the model used the same mechanism (telescopic leg actuators) for all conditions, it spontaneously discovered an optimal walking gait at slow speeds and an optimal running gait at high speeds. It also discovered a hybrid pendular-running gait at intermediate speeds. Although humans do not naturally employ pendular-running locomotion, evidence that various avian species use a similar pattern have since been described (Usherwood, 2010).

Here we employ a similar model, also with two massless telescopic leg actuators and a point mass body (Figure 7A). The equations of motion are detailed.

(10) $$m_{c}{\ddot{x}}_{c}=\sum_{\left(l,r\right)}F_{i}\left(\frac{x_{c}-x_{fi}}{L_{i}}\right)$$

(11) $$m_{c}{\ddot{y}}_{c}=\sum_{\left(l,r\right)}F_{i}\left(\frac{y_{c}}{L_{i}}\right)-m_{c}g$$

where ${\ddot{x}}_{c}$ is the horizontal acceleration of the CoM, $F_{i}$ is the leg actuator force for both left (***l***) and right (r) legs, $x_{f}$ is the position of the foot contact (where the force vector originates from; the foot contact is a constant parameter since a non-slip contact is assumed) for both legs and L is the effective leg length of each limb, as formulated below.

(12) $$L=\sqrt{{\left(x_{c}-x_{f}\right)}^{2}+y_{c}^{2}}$$

In order to ensure the model does not take advantage of unreasonable leg length values (e.g., $L\gg d_{s}$), a path constraint was applied to the optimization. The constraint mandates that a leg actuator cannot produce force if the CoM is further away from the foot contact than the maximum leg length indicates.

(13) $$F_{Leg}\left(L_{max}-L\right)>0$$

A control optimization protocol was applied (as described in the single actuator methods) that included a work-based cost and a force-rate-squared cost for each leg actuator (Eq. [8]). The force-rate-squared term serves to penalize highly impulsive forces in favor of more realistic, smooth leg forces. The mechanical power of the leg actuators (${\dot{W}}_{leg}$) utilized in the cost function is shown as a function of leg force ($F_{leg}$) and leg length velocity ($\dot{L}$).

(14) $${\dot{W}}_{Leg}=F_{Leg}\dot{L}$$

(15) $$\dot{L}=\frac{\left(x_{c}-x_{f}\right){\dot{x}}_{c}+y_{c}{\dot{y}}_{c}}{L}$$

GRF of this model are shown in comparison to empirical data (Figure 7B). Many key features of human walking are reflected in the vertical GRF of the model. For example, the model oscillates between periods of single stance (a single leg provides force) and double stance (both legs provide simultaneous force). The characteristic double-humped profile is also notable in the optimal solution of the model. The hump towards the end of stance occurs due to active extension of the trailing leg and replicates the preemptive push-off found in human walking. Recall, the preemptive push-off does positive work to reorient the CoM velocity vector more perpendicularly to the force vector of the coming collisional impulse at heel strike (Figures 4B and 7C,D). This impulse manifests in the signal as the hump at the beginning of the next stance leg and occurs due to extension forces of the forward leg resisting compression. Similar to human walking, this sequencing helps to maintain momentum with minimal loss at the step-to-step transition. Horizontal GRF are also similar—both showing a deceleration phase towards the beginning of stance and an acceleration phase towards the end of stance. Finally, the point of zero horizontal acceleration occurs approximately at mid-stance (CoM is above the foot contact position).

Overall, the optimal solution of this model takes advantage of the passive dynamics of the inverted pendulum during the majority of single stance by holding a rigid leg (constant radius trajectory means the leg does not extend, and this has no work-based cost since leg velocity is zero). However, the model deviates from this pattern at the step-to-step transition and relies on impulsive forces by both legs simultaneously in order to manage the redirection of the CoM from down to up. The majority of the model’s work-based cost is accumulated at this transition, however, it is managed as efficiently as possible, short of using ideal impulses (recall these solutions are penalized by a force-rate-squared cost for more realistic force profiles).

### 3.2.2. Forced Coupled Oscillator Model (No Actuator Cost)

The inverted pendulum with telescopic leg actuators is arguably the most realistic model for human walking, as compared to other walking mechanisms described in the contribution thus far. This is because previous models considered rigid strut-like legs (as well as, in one case, collapsible legs) and relied on a fixed-orientation actuator to provide force directly to the CoM. However, humans use legs themselves as actuators (non-fixed orientation) to apply force to the body. Still, it may be useful to consider a composite of the two strategies, where a total of three actuators are available to the model: two telescopic legs plus an additional vertical force applied directly to the CoM. Essentially, this allows the model to deconstruct the GRF into distinct signals that are distributed among the different actuators, thereby implying optimal function based on the orientation and magnitude of the resulting force vectors.

**Figure 8:**
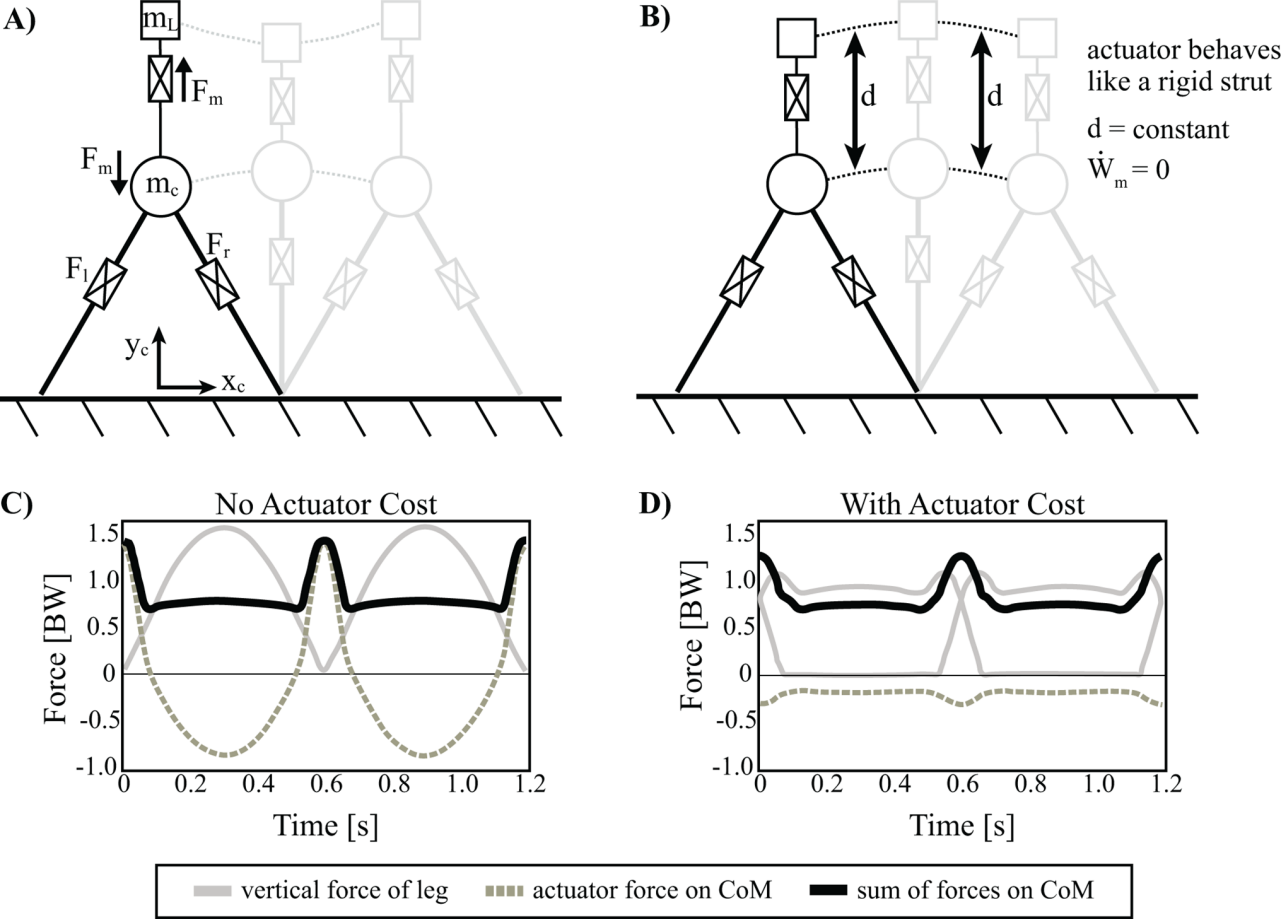
Inverted pendulum model with telescopic leg actuators and a coupled oscillator at the body center of mass. Point mass trajectories of body ($m_{c}$) and load ($m_{L}$) are shown for the optimal solution where **(** **A)** actuator cost *is not* considered and **(** **B)** actuator cost *is* considered. **(** **C** **)** Ground reaction forces are shown for the solution when actuator cost *is not* considered and **(** **D)** for when actuator cost *is* considered. The sum of forces on $m_{c}$ are similar, but its components are distributed over all three actuators. It should be noted that the vertical range of the load's trajectory in pane (A) is scaled down for illustrative purposes.

Specifically, a coupled oscillator mechanism is used to consider a more specific form of actuator force applied to the CoM. The coupled oscillator mechanism consists of a linear actuator that drives a point mass ($m_{L}$) in vertical oscillations off the body (Figure 8A). The influence of these forces is manifested through the reaction force of the actuator on the body CoM ($m_{c}$). In this model, the added point mass of the coupled oscillator mechanism can be thought of in two ways: (1) as an additional load that the walking mechanism carries or (2) as a portion of the existing CoM now split into two pieces (in either case, $m_{L}<m_{c}$). Although this distinction does affect force magnitudes, we account for this by reporting forces in units of body weight, where $1\;BW=g(m_{c}+m_{L})$. This is analogous to a horse’s head bobbing up and down during locomotion. The mass of the head is a portion of the total body weight and the neck muscles are the actuator to help drive (and control) this load, although in this case much of this oscillation is likely passively managed by the complex nuchal ligament (Gellman and Bertram, 2002). Regardless, the oscillation of the head is thought to have an impact on the whole-body locomotion of the animal, as the head typically makes up about 10% body mass.

Additional constraints are modeled such that the load is driven with a continuous periodic motion—the consequence of which is an average actuator force equivalent to the load’s weight. With these constraints, the actuator is prohibited from merely performing Groucho patterns (constant vertical force to the CoM) like the single actuator design described previously. This is because the constant reaction force required to bear CoM weight would result in an equal and opposite force accelerating the coupled load in the downward direction for the duration of the step, making a periodic pattern infeasible. Instead, the actuator force must provide equal amounts of positive and negative work to maintain steady state kinematics of the load. Since an average upward force is required for the actuator to maintain full support of the load’s weight, a constant loading effect is felt (in the downward direction) at the CoM of the walker, in addition to the dynamic oscillation force.

The equations of motion of the previous model are expanded to include the forces imparted by the coupled oscillator mechanism.

(16) $$m_{t}{\ddot{x}}_{c}=\sum_{\left(l,r\right)}F_{i}\left(\frac{x_{c}-x_{fi}}{L_{i}}\right)$$

(17) $$m_{c}{\ddot{y}}_{c}=\sum_{\left(l,r\right)}F_{i}\left(\frac{y_{c}}{L_{i}}\right)-F_{m}-m_{c}g$$

where $m_{t}$ is the total system mass ($m_{c}=0.8m_{t}$ and $m_{L}=0.2m_{t}$) and $F_{m}$ is the optimized force of the coupled oscillator actuator. Equations describing motion for the added point mass, $m_{L}$, are shown below.

(18) $$m_{L}{\ddot{x}}_{L}=m_{L}{\ddot{x}}_{c}$$

(19) $$m_{L}{\ddot{y}}_{L}=F_{m}-m_{L}g$$

Furthermore, leg length and mechanical power of the leg actuators (as well as the maximum leg length constraint) are implemented per equations [12-15].

First we consider the optimal solution for the model described with no actuator cost (i.e., work done by the coupled oscillator actuator imparts no cost influence on the optimal solution, however work done by the leg actuators is considered) (Figure 8A,C). In this case, the GRF shows a prominent single hump, as opposed to the more typical double-humped profile observed in the model without the coupled oscillator. Essentially, the legs provide isometric, weight-bearing forces (body plus average loading of coupled oscillator) during the stance phase of the gait while the third actuator takes over forces that facilitate mechanical work done to redirect the CoM near the step transition. The summation of the leg actuator and the coupled oscillator force profiles replicates the summed forces of the familiar double-humped pattern, which is responsible for bearing body weight and oscillating the body (inertial force) (Figure 8C). In many ways the solution is unsurprising given that the double-humped profile is already known to be an optimal pattern. The only difference is that the optimization spontaneously seizes on a strategy that delegates the energetically expensive work-based portion of the force profile to the actuator (since there is no cost penalty to do so) and the legs maintain the inverted pendulum portion of stance since these forces are largely isometric (i.e., constant leg length with zero work done).

### 3.2.3. Including Actuator Cost

The coupled oscillator model described above requires essentially no work of the telescopic leg actuators. As such, it is a passive gait, from the perspective of the biped since the leg actuators are used mostly as rigid struts. However, it is useful to consider whether there is any utility in the coupled oscillator strategy beyond the supplementation of free mechanical work available via the coupled oscillator actuator. Therefore, the same model is used to consider an optimal solution that seeks to minimize actuator work in the coupled oscillator as well as work done by the legs. Additionally, a force rate penalty is utilized for all three actuators to avoid unrealistic impulsive forces. The equation for actuator work is listed below, and the resulting optimal solution is shown in Figure 8B,D.

(20) $${\dot{W}}_{m}=F_{m}\dot{d}$$

(21) $$\dot{d}={\dot{y}}_{L}-{\dot{y}}_{c}$$

The solution looks quite different from that which neglected the coupled oscillator actuator cost. Instead of the actuator providing dramatic sweeping impulses to the load/CoM system, it acts like a rigid strut. The force oscillations observable in Figure 8D facilitate a kinematic trajectory that changes in tandem with the body point mass. As a result, the displacement between the two point masses (d) is constant, and the relative velocity ($\dot{d}$) is zero. Thus, the actuator is not used to perform mechanical work (Figure 8B). Indeed, the cost of this solution is the same as the model with no coupled oscillator (Table 1). Ultimately, this result suggests that the coupled oscillator actuator cannot reduce the cost of the overall system, even though it has already been shown capable of reducing leg work. In order to understand why this mechanism cannot reduce the cost overall, the apparent cost of the actuator was manipulated. Specifically, a weighting coefficient, $C_{m}$ was introduced in order to discount the cost of the coupled oscillator actuator’s mechanical power in the objective function during optimization.

**Table 1:** Cost summary for models. The non-dimensional work is shown for all relevant actuators (legs, actuator at the center of mass and total). Work is indicated with not applicable (“na”) if the particular model does not include such an actuator.

**Model Description**	**Leg Work (** **×** **10^−2^)**	**Actuator Work** **(**×**10^−2^)**	**Total Work (**×**10^−2^)**
Horizontal Force	na	22.05	**22.05**
Telescopic Legs	6.25	na	**6.25**
Telescopic Legs + Coupled Oscillator	0.29	301.25*	**301.54**
Telescopic Legs + Coupled Oscillator	6.25	0	**6.25**
Telescopic Legs + Coupled Oscillator	5.21	2.32*^†^*	**7.53**

*Cost of actuator work is not considered for this optimal solution.

^†^Cost of actuator work is not considered for this optimal solution but actuator constraints on stroke, force capacity and voltage supply are implemented.

(22) $$C_{m}{\dot{W}}_{m}=C_{m}F_{m}\dot{d}$$

$$0<C_{m}<1$$

**Figure 9:**
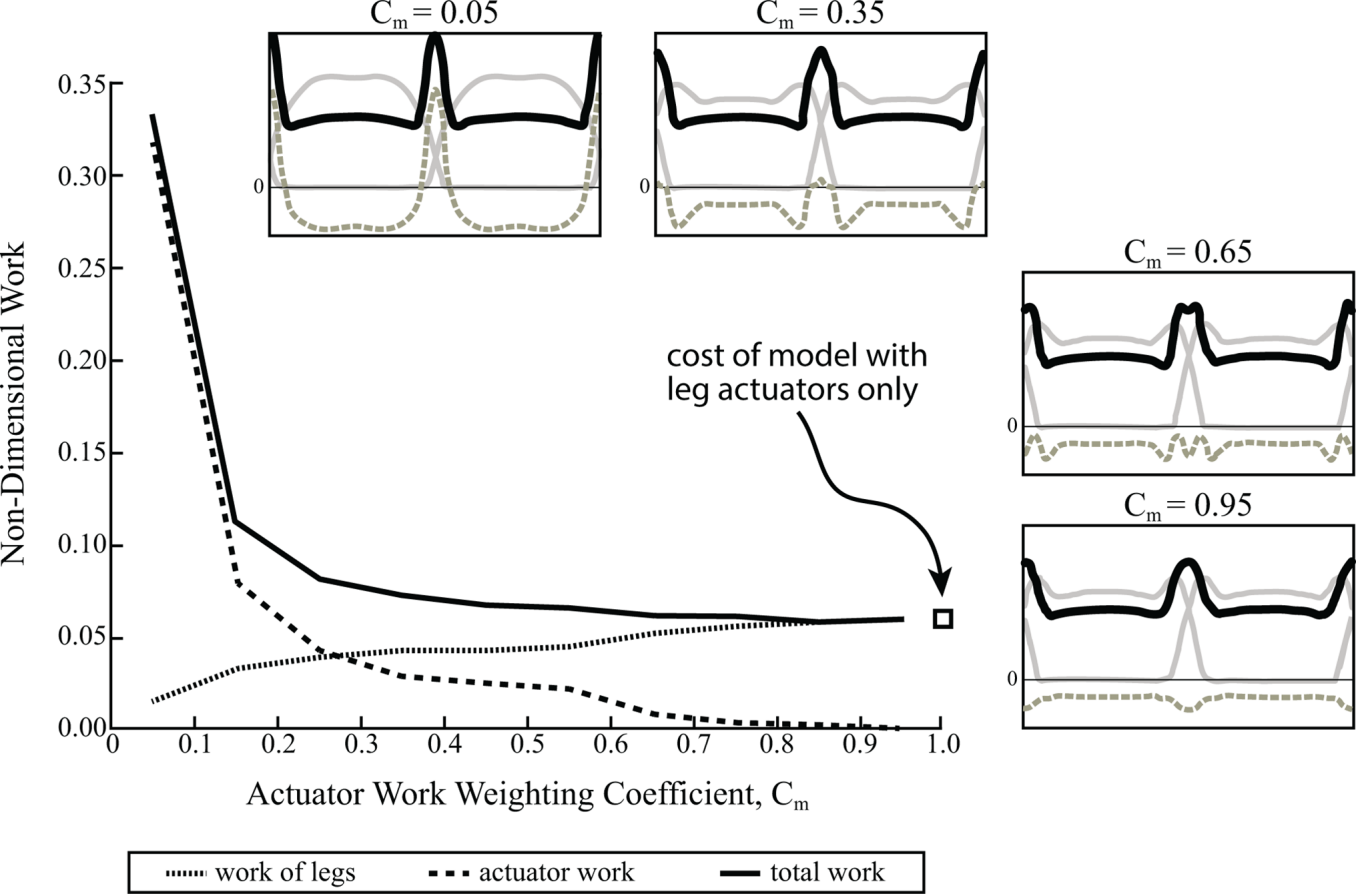
Non-dimensional mechanical work is shown for contribution of legs and coupled oscillator actuator, as well as total work over a range of weighting coefficients ($\;0<C_{m}<1\;$). This reduces the apparent cost of the actuator and thus, alters the optimization solution. Actuator work contributes most of the work for a low coefficient and almost no work for a high coefficient. The opposite is true of the legs. Ground reaction force profiles are shown for four different weighting coefficients (solid grey lines are single leg forces, dashed grey lines are actuator reaction forces and solid black lines are a summation of both). The total work of the model approaches the work done by the model with no coupled oscillator actuator at higher coefficients.

By implementing a weighting coefficient, the energetic benefit of the actuator’s force oscillations is less obscured by its diminished cost, allowing suboptimal solutions to be evaluated. Figure 9 shows the full work (i.e., no discount) done by the actuator, as well as the leg work and total work of the legs plus the actuator over a range of weighting coefficients. Force profiles for optimization solutions are also shown (same format as in Figure 8C,D) for the following weighting coefficients: $C_{m}=0.05,0.35,0.65\;and\;0.95.$ As expected, the force profiles are very similar to the case of no actuator work considered when $C_{m}=0.05$. However, the force oscillations become less pronounced at higher $C_{m}$ values, until they begin to converge on a rigid strut solution when $C_{m}=0.95$ (Figure 9).

At very high discounts ($C_{m}=0.05$) the full actuator work increases drastically, although leg work is greatly reduced. At low discounts ($C_{m}=0.95$) and even moderate discounts, total work plateaus to the cost of the model with no coupled oscillator, while actuator work diminishes and the legs take up more and more of the cost. Essentially, the energetic advantage that the coupled oscillator actuator provides to the legs is overshadowed by its full cost, and as a result, the optimal solution uses the actuator as a rigid strut (no work), unless its cost is artificially discounted.

It is perhaps surprising that the addition of a coupled oscillator actuator cannot improve upon the energetics of a bipedal mechanism without it. Indeed, the step-to-step transition is costly in part because the orientation of the legs during double stance means that both positive and negative work must be done simultaneously on the body in order to redirect the CoM trajectory (Donelan et al., 2002a, Donelan et al., 2002b). The non-vertical orientation of the legs (in contrast to the vertical actuator) means that a larger force magnitude—and consequently, more work—is required to alter the body trajectory from downward to upward.

Although it is unclear exactly why work of the actuator is more expensive than the work it saves the legs, there are a few identifiable factors that contribute to its cost. First, in order to offload the legs during their high mechanical power at double stance, the load must be accelerated downward to incite a positive reaction on the body, and this incurs a cost. Next, this action must be paid back with positive acceleration in order to maintain a positive/negative net work balance (this is required to have a steady state, repeatable pattern). Ideally, the positive acceleration (negative reaction force) can be supported by the legs with isometric force during single stance (i.e., no extra energetic cost to the legs), however actuator work is still required to brake the load from its acceleration and then lift it up against gravity. The consequence of these factors and their interactions is such that any use of the actuator (beyond isometric force) costs more than it saves.

Ultimately these results indicate that the economy of a walking machine would not benefit from the implementation of a coupled oscillator mechanism as described. Still, the concept may retain its utility in a system where reducing leg work (rather than work overall) is desirable.

### 3.2.4. Applying Realistic Actuator Constraints

One way to translate the coupled oscillator model into a real-world context is to imagine a human walking with such a mechanism mounted to a body harness. Although this design concept would not benefit the energetics of the whole system (person plus machine), it could still prove a useful strategy for reducing leg work and mechanical power required by the person to walk.

In this example, two linear shaft motors (model: S320T, Nippon Pulse America Inc., Radford, Virginia) are used. The two motors are controlled to act in unison and with a parallel configuration (one mounted anterior to the torso and the other mounted posterior to the torso). The summed effect of the two motors embodies the theoretical actuator allowing known loads with vertical oscillations to apply impulses to the CoM (front and back actuators are used to minimize pitch moments since the harness can only be mounted at the surface of the torso, a small moment arm distance from the true CoM). Similar to the model, reaction forces of the permanent magnets (mounted to the frames) are felt by the user’s body through the attaching harness. It is hypothesized that an individual will choose motor patterns based on the principle of energy minimization, in which, the optimal work-based solutions discovered by the optimal control problem reflect the coupled oscillator interaction chosen. Although current literature suggests that humans sometimes adapt gait patterns to accommodate elastic load oscillations to reduce metabolic exertion (Rome et al., 2005, 2006; Castillo et al., 2014; Ackerman and Seipel, 2014), more evidence is needed to show that humans can employ energy minimization strategies consistent with the interactions proposed by the forced coupled oscillator mechanism described. Still, realistic system constraints and considerations can be implemented for the applied problem.

In order to consider the dynamics of the actuators in this applied system, the variable $F_{m}$ is updated.

(23) $$F_{m}=K_{F}i_{a}-c_{d}\dot{d}$$

where $i_{a}$ is the armature current, $K_{F}$ is the motor force constant that relates current and force and $c_{d}$ is the damping coefficient that characterizes viscous damping of the motor.

Three additional constraints are implemented to simulate a more realistic system: (1) motor/load kinematic oscillation range is limited by stroke; (2) maximum force capacity is limited by the motors; (3) maximum voltage is limited by a direct current power supply (model: PS16L80, Advanced Motion Controls, Camarillo, California). These constraints are described mathematically.

(24) $$-\frac{S}{2}\leq d\leq \frac{S}{2}$$

(25) $$-F_{m,max}\leq F_{m}\leq F_{m,max}$$

(26) $$-V_{PS,rms}\leq V\leq V_{PS,rms}$$

where d is the displacement of the load relative to the body point mass ($d=y_{L}-y_{c}$), S is the motor stroke, $F_{m,max}$ is the maximum acceleration force, $V_{PS,rms}$ is the root-mean-square voltage available from the power supply and V is the total voltage draw, determined from Kirchhoff’s Voltage Law.

$$V=V_{Ri}+V_{emf}+V_{ind}$$

where $V_{Ri}$ is the voltage at the armature resistance, $V_{emf}$ is the voltage due to back electromotive force (emf) and $V_{ind}$ is the voltage due to inductance. By assuming that force is proportional to current and noting Ohm’s Law, we derive:

(27) $$V=\frac{R_{a}}{K_{F}}F_{m}+K_{emf}\dot{d}+\frac{L_{ind}}{K_{F}}{\dot{F}}_{m}$$

where $R_{a}$ is the armature resistance, $K_{emf}$ is the motor back emf constant, and $L_{ind}$ is inductance. Note that a motor controller is chosen specifically for this system (model: DMC4123, Galil Motion Control, Inc., Rocklin, California) with sinusoidal amplifiers (D3520), however actuation performance is not further limited since constraints of the other equipment are more restrictive.

**Figure 10:**
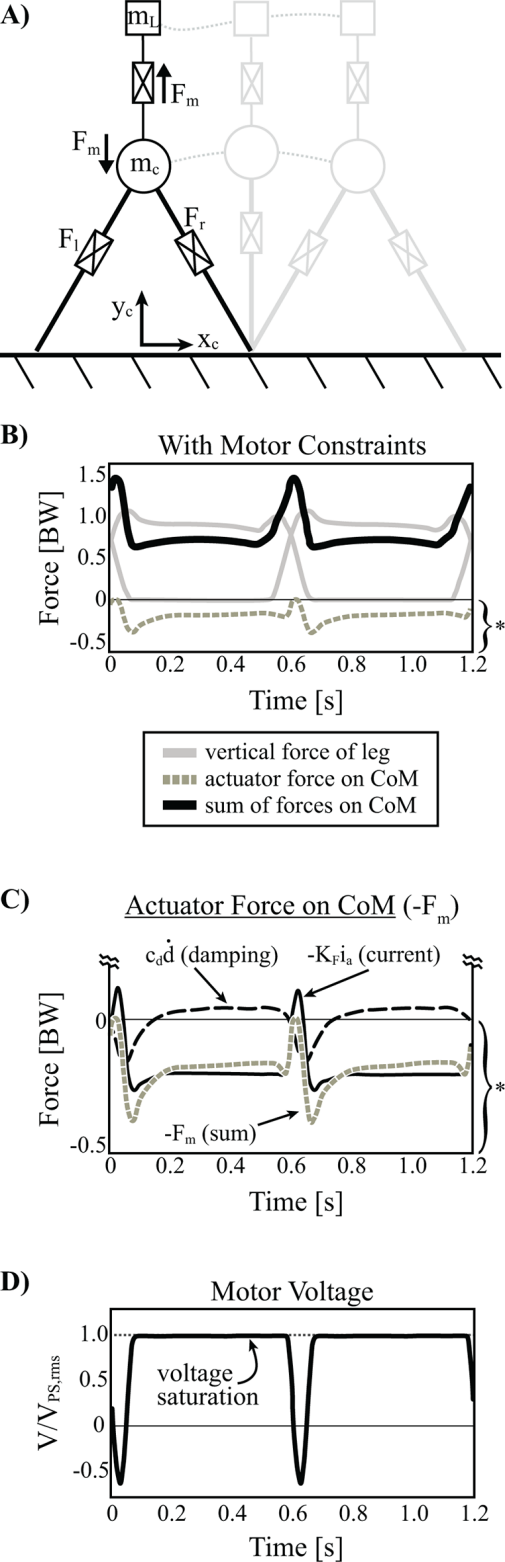
Inverted pendulum model with telescopic leg actuators and a coupled oscillator at the body center of mass. **(** **A** **)** Point mass trajectories of body ($m_{c}$) and load ($m_{L}$) are shown for the optimal solution where the actuator cost is not considered. However realistic actuator dynamics (damping) and constraints (e.g. stroke, motor force capacity, peak voltage available from power supply) are implemented. **(** **B** **)** Vertical ground reaction forces are shown for individual legs, the total actuator reaction force on the center of mass (damping plus force due to armature current) and the summation of forces. Note that the grey dashed line is the same as in **(** **C)** where both terms of actuator reaction force are shown (damping and force due to armature current). Note, the actuator reaction force and its components are scaled by the bracket and asterisk indicated at the bottom right of pane (B)**. (** **D)** Armature voltage normalized to maximum voltage available from the power supply is shown since this is the only restricting actuator constraint affecting the solution.

When the actuator dynamics and constraints are implemented, the optimization converges on a solution that utilizes a positive pulse of motor reaction force applied to the body (negative force on the load) near the middle of double stance (where maximal leg power is produced; Figure 7D, Figure 10B,C). Essentially, this allows for redirection of the CoM while the load is effectively weightless (i.e., $F_{m}\approx 0$), from the perspective of the legs. However, this offloading must be paid back in order to maintain a steady state pattern and so a negative reaction soon follows. The sequencing is beneficial overall since the positive pulse helps to offload the legs during a time of high mechanical power output (near the middle of double stance) and the negative pulse hinders the legs during a time of diminished mechanical power (closer toward single stance). It should also be noted that much of the negative pulse is provided by damping force (and some armature current) since load velocity peaks shortly after the positive pulse (~90° phase delay; Figure 10C).

During single stance, the total reaction force of the motor is near the weight of the load, due mostly to the armature current (although some damping force is present). The effect of this force during single stance does not contribute much to the cost of the solution since mechanical power is largely zero due to the constant leg length (inverted pendulum strategy). However, leg force decreases slightly over stance in an asymmetrical pattern as it provides isometric weight-bearing force that is offloaded slightly by an increasing damping force (Figure 10B,C). This damping occurs due to the body CoM slowing its vertical motion relative to the load as it rises to mid-stance and then begins to fall away from the load. This pattern of reaction force continues into the beginning of push-off and helps to unload the legs slightly during this time. Eventually the positive pulse of reaction force occurs again at the next step and the cycle repeats.

Given that the actuator system provides beneficial offloading to the legs at a time when total motor voltage is not nearly saturated (~67% $V_{PS,rms}$; Figure 10D), it is fair to question why higher force magnitudes are not used. However, the positive pulse must be paid back with negative reaction force (positive force on the load) and the maximum voltage becomes saturated at forces just beyond the weight of the load (Figure 10D), leaving little room for additional oscillation. In fact, the maximum force allowed by the system can be calculated as follows (assuming $\dot{d}=0$, ${\dot{F}}_{m}=0$):

(28) $$F_{m}\left(V=V_{PS,rms}\right)=\frac{K_{F}}{R_{a}}V_{PS,rms}$$

With the parameters of the system selected, maximum force production is limited to approximately 115% the weight of the load. This limitation comes from the voltage available from the power supply rather than the force capacity of the motors themselves. In fact, the motor force itself only ever approaches about 33% of the motor force capacity, and as such, this constraint does not limit the solution. Likewise, the maximum stroke range used in the solution is around 22% of that available, and so this constraint also does not limit the solution.

Given that the actuator system is heavily restricted in its ability to pay positive reaction forces back with negative forces beyond the weight of the load, it must rely heavily on the damping force that dominates immediately following the positive pulse. As well, the motor can provide some additional negative force beyond the voltage limitation at this time since the back emf voltage reduces the overall voltage draw.

The strategy just outlined reduces the leg work accumulated over a step, even with the limitations of the actuator constraints. However, the overall system expends more work in total, since the actuator strategy is more expensive than the savings it provides to the legs (Table 1). Still, if the design goal of such a device is to offload the leg work done by a human wearing an exoskeleton, then the solution presents this potential.

## 4. Other Considerations

### 4.1. Leg Swing Dynamics

The reader may have noticed that the complication of leg swing dynamics has not played a formative role in the development of walking models discussed here. Although this is an important aspect of locomotion that ultimately cannot be ignored, we have chosen to focus on the underlying mechanisms that have a dominant influence on the energetics of whole-body trajectory management (Donelan et al., 2002a; Kuo et al., 2005). There is some evidence that swinging the leg consumes approximately 10–33% of metabolic expenditure during bipedal walking (Doke et al., 2005; Gottschall and Kram, 2005; Umberger, 2010), however the dynamics of a pendular leg (or more specifically, a double pendulum) can likely be facilitated with mostly passive dynamics.

For example, a slightly more complex and more thoroughly actuated model replicating human gait (Hasaneini et al., 2013) spontaneously employs a bang-coast-bang strategy to power leg swing in walking. Specifically, a quick burst impulse is used to accelerate the leg forward (first bang), then the leg swings with mostly passive dynamics (coast) and another quick burst impulse is used to decelerate the leg before the next touchdown (second bang). It has previously been recognized that similar activation patterns govern natural leg swing in humans (Mochon and McMahon, 1980; Doke et al., 2005; Doke and Kuo, 2007). Furthermore, the bang-coast-bang strategy has generally been demonstrated an optimal mode of movement control when initial and final conditions require a similar state (e.g., initial velocity equals final velocity) (Srinivasan and Ruina, 2006; Srinivasan, 2011).

The mechanical cost of a bang-coast-bang leg swing is proportional to the leg’s rotational velocity squared, given that the impulse must do work to impart kinetic energy ($W=\frac{1}{2}I\omega^{2}$) for a desired travel of the leg over the duration of swing (Srinivasan, 2011). Ultimately, the rotational velocity of the leg is related to the stride length that the foot must sweep through and the time duration of the swing. Assuming that double stance is relatively short, it then follows that the time duration of swing is approximately equal to step frequency. Thus, step length and step frequency should play an important role in determining the energetic cost of leg swing. Walking is associated with relatively low speeds (i.e., low step frequency and step length), and so it is predicted that the leg swing cost should also be low, as compared to other gaits such as running. In addition, step frequency and step length were constrained to the same values in all models (see Eq. [7]), and as such, there is likely a general increase of the cost surface for solutions presented here. However, the unaccounted cost of leg swing should not change the optimal solutions presented, since a global shift in the cost surface does not change its shape nor the location of the minimum.

It should be noted that this speculation assumes a decoupling between the leg dynamics and the rest of the body. However, it is easy to imagine that oscillations from a coupled oscillator mechanism, for example, may have an influence on the passive nature of the double pendulum leg, and thus, a more complicated energetic interaction. More detailed and thorough models should be developed to answer such questions about the energetics of leg swing and determining interactions.

### 4.2. Mechanical Work, Metabolic Energy and Electrical Power Consumption

All of the models presented here utilize a mechanical work-based cost for optimization. Although it is ultimately the metabolic energy that most likely influences motor control choices regarding movement patterns in humans, a work-based cost was chosen instead. For one, work is easily quantifiable as a mechanical variable, whereas metabolic energy requires the consideration of a more complicated physiological interaction. For example, the metabolic energy associated with isometric contraction (no work) is costlier for force generation than it is for force maintenance (Russ et al., 2002).

A simple approximation of the metabolic energy associated with work done by the muscles is determined by considering the differential efficiency of muscle contraction (25% for concentric contraction and −120% for eccentric contraction). However, given that only steady state gaits were considered by the optimizations, equal amounts of positive and negative work must be done over a step. Thus, the differential conversion from work to metabolic energy should not change any of the optimal results, other than the overall value associated with cost.

Also, since the models are meant to represent theoretical walking mechanisms that can be thought of as either robots or simple abstractions of human bipeds, it is unclear that metabolic cost is even the most appropriate cost to consider. Given that different actuators consume energy in different ways, it seems appropriate to consider mechanical work, since it is a physical requirement that all actuators must consume at least this energy (biological or artificial). An electromagnetic shaft motor was considered for implementation in the coupled oscillator mechanism, and as such, electrical power could have been used for the optimization. However, this cost scales somewhat differently from simple mechanical work, and so this changes the cost scaling comparisons of the leg actuators relative to the oscillator motor. Consequently, mechanical work was used as a more generally comparable energetic cost.

## 5. Cost Results Summary

In this contribution, we have outlined multiple reductionist walking mechanisms. Although each model is limited by the inherent physics of its individual makeup, they all test the employment of strategies reflecting one or more principles important to efficient bipedal locomotion. Although the single actuator Groucho design allows for zero work to be done over a step, this mechanism represents a trivial solution, which is already epitomized by wheeled mechanisms, and these systems have their own considerations less relevant to truly legged machines (e.g., typically requires some form of infrastructure, such as a road, since the effective radius is invariant). The horizontal actuator inverted pendulum model utilizes a bang-coast-bang approach in order to ensure continuous periodic motion of the CoM, however this model imparts a large cost on the actuator, since it must provide impulses to slow the CoM to a full stop and reaccelerate up to speed with every step. The sequencing of positive and negative work is restricted to operate suboptimally (effective heel strike before push-off) simply because a resting motion is necessary at the step-to-step transition. The energetic cost of this model is unnecessarily excessive relative to more economic designs discussed thereafter ($cost≅22.05x10^{-2}$; Table 1).

The inverted pendulum with telescopic legs represents a model that can replicate dynamics more similar to human walking. The total cost of the leg actuators is approximately 3.5 times less than the fixed-horizontal actuator model ($cost≅6.25x10^{-2}$; Table 1), even though it has twice the number of actuators. This result is largely due to the extra degree of freedom given to the CoM so it can deviate from a constant radius profile. This is important because it allows for a continuous periodic gait pattern that maintains momentum (minimizes leg work) at the step-to-step transition rather than bringing the system to rest with every step. Still, the orientation of the legs at this transition (non-vertical) also exists as a limitation to what is possible for energy minimization, since positive and negative work of each leg must be done simultaneously, and this is somewhat wasteful.

The coupled oscillator mechanism is used to take advantage of inverted pendulum motion during stance and vertical actuation at the step-to-step transition. When the cost of the coupled oscillator actuator is not considered, it completely takes over the expensive portion of the gait required for redirecting the CoM motion from downward to upward, and only uses the legs to bear isometric loads with mostly zero leg deflection during single stance (almost no work in this portion). The cost of the legs is essentially null in this model however the work done by the coupled oscillator actuator is prohibitive ($cost≅301.25x10^{-2}$; Table 1).

When the cost of the coupled oscillator actuator is considered, the optimization converges on a strategy that uses the actuator for isometric force production only. This is the dynamic equivalent of returning the load mass back to the CoM, and exists essentially as a null result. The optimal pattern exists as it does because the cost of using the actuator to perform work is costlier than not using it at all. The resulting cost is equivalent to the model with no coupled oscillator actuator ($cost≅6.25x10^{-2}$; Table 1).

Finally, the coupled oscillator model is optimized with no actuator cost, but with more realistic system dynamics and constraints deemed potentially restricting from data sheets of commercially available equipment. The resulting optimal pattern utilizes impulsive forces to reduce the weight of the load at costly double stance. It also takes advantage of damping forces to help oppose the relative acceleration of the load over the duration of single stance. This results in a ground reaction force, which is somewhat asymmetrical. The overall cost of the model is approximately 18.9% higher than with no coupled oscillator ($cost≅7.43x10^{-2}$; Table 1), however leg work is still reduced by about 16.6% ($leg\;work≅5.21x10^{-2}$; Table 1).

## 6. Models and Their Solutions in Context

We began this contribution by recognizing an alternate definition for the fundamental task of locomotion as the optimal dynamic interaction between the system mass and the external environment as mediated by mechanisms available to the organism (Figure 1). Most exoskeleton designs tend to focus on principles directed at specific mechanisms of gait. For example, a variety of active ankle exoskeletons have been developed in recent years with the strategy of providing mechanical power directly at the ankle joint during push-off and have achieved successful reductions in metabolic consumption ranging from 6–24% the cost of unassisted walking (Sawicki and Ferris, 2008; Malcolm et al., 2013, 2014; Zhang et al., 2017). Although this approach clearly has potential for success when the mechanism of focus is well understood in the context of its role in whole-body energetics, a different approach is to consider strategies that influence the interaction between the organism and its environment more directly.

Indeed, we began this discussion by entertaining the notion that the leg actuators in Collins et al.’s variation on the passive dynamic walker (Collins et al., 2005) could mostly be replaced with a single actuator at the CoM. The dialogue that followed eventually culminated in the coupled oscillator exoskeleton as a more elaborate manifestation of this approach to control an optimal interaction at the body more directly. Even though the resulting optimal strategy turned out to be similar (apply impulsive forces to the body near push-off), we have shown that this type of actuation does not necessarily need to be applied at the ankle joint, at least in theory. This is an important insight given that carrying loads (e.g., actuator, transmission, battery, etc.) at the foot can result in a cost increase 4.4 times greater than carrying the same load at the waist and 1.7 times greater at the shank or thigh (Browning et al., 2007). Furthermore, the coupled oscillator strategy does not seek to minimize loading (as an ankle exoskeleton might), but rather *requires* some loading to operate. As such, the weight of the actuator, transmission, battery, etc. actually helps to generate the reaction forces that benefit leg work. In fact, increased loading could potentially minimize the necessary stroke required, assuming that voltage constraints are improved over the power supply currently suggested in the model. Of course, empirical studies are still needed to verify the theoretical potential of a coupled oscillator exoskeleton in practice.

Overall, we view the control optimization models discussed here as a direct exploration of how the interaction (system mass and external environment) can be optimized and to what extent. Although focus is directed at the optimal interaction and not at the mechanism, it is impossible to facilitate the interaction in the absence of a mechanism. As such, we rely on reductionist abstractions of real mechanisms. For example, biological legs with sophisticated musculature and joint spaces are collapsed into simple telescopic actuators that can actively extend. Electrical windings and ferrous shafts mounted to body harnesses are replaced with an extensive actuator driving a point mass load. Although some may view these simplifications as inaccurate depictions that do a disservice to complex systems in real life, the reductionist nature of such mechanisms allows for clearer interpretation of what makes an interaction optimal in the first place (i.e., less moving parts).

This is not to say that the details of a mechanism are not important. To the contrary, appropriate tuning of mechanisms (e.g., spring stiffness), for example, can greatly affect the performance of an exoskeleton (Sawicki and Khan, 2016). However, the design process of such devices is well-served by a prior understanding of its effect on energetic exertion at the whole-body level (assuming this is the goal), before focusing on such details as tuning. This is arguably validated by the fact that ankle exoskeletons have likely benefitted from the prior understanding of the importance of push-off on the energetics of human walking.

To some degree, the practice of reductionist actuation modelling may be interpreted as an arbitrary thought experiment. However, we maintain that each variation of the bipedal walker is a new opportunity to gather insight on the fundamental barriers to efficient actuation in locomotion. The results of such practice—if interpreted carefully—can lead to important advances in the perspective that roboticists and biologists hold on the science of animal and machine locomotion.

## Author Contributions

JB conceptualized the manuscript and performed literature review. RS developed mathematical derivations of the models and performed all optimization analyses in MATLAB. Both RS and JB contributed to the composition and editing of the manuscript.

## Conflict of Interest Statement

The authors declare that the research was conducted in the absence of any commercial or financial relationships that could be construed as a potential conflict of interest.
